# Secondary myelodysplastic syndromes identified via next-generation sequencing in a non-small cell lung cancer patient

**DOI:** 10.1186/s12920-021-01147-y

**Published:** 2021-12-20

**Authors:** Yongzhi Feng, Xialin Chen, Keran Jiang, Ding Zhang, Feng Tao, Dan Ni, Jun Zhang, Lixin Wu, Jinping Cai, Libin Jiang, GenHua Yu, Lin Shi

**Affiliations:** 1 The First Hospital of Jiaxing, Jiaxing, China; 2 Shaoxing People’s Hospital, Shaoxing, China; 3 3D Medicines Inc, Shanghai, China; 4 The Second Affiliated Hospital of Jiaxing University, Jiaxing, China; 5 Zhejiang Rongjun Hospital, Jiaxing, China; 6 Zhejiang Hospital of Traditional Chinese Medicine, Hangzhou, China; 7 Zhebei Mingzhou Hospital, Huzhou, China

**Keywords:** Myelodysplastic syndromes, Non-small cell lung cancer, Next-generation sequencing, Copy number variations, Case report

## Abstract

**Background:**

Myelodysplastic syndrome (MDS) is a group of clonal disorders characterized by ineffective and dysplastic hematopoiesis in the bone marrow with a high risk of progression to leukemia. Many studies have demonstrated that chemo-radiotherapy for cancer patients and exposure to certain chemicals may increase the risk of secondary MDS, which is characterized by specific chromosomal abnormalities and genomic alterations. Since next-generation sequencing (NGS) has been widely used for the diagnosis of cancer patients, advanced analysis of the sequencing data may provide supplementary information for secondary MDS.

**Case presentation:**

A male patient with non-small cell lung cancer (NSCLC) and bone metastases has presented distal obstructive inflammation, the enlargement of the left hilar, mediastinal lymph node metastases, and multiple bone metastases. This patient has undergone long-term exposures to certain chemicals. Moreover, the deletion of chromosome 7 and 5q is detected in his peripheral blood sequencing, indicating secondary MDS, subsequently confirmed by bone marrow examination.

**Conclusion:**

In this case, an NSCLC patient was diagnosed with secondary MDS via NGS analysis, indicating that the NGS analysis may serve as supplementary for diagnosis of secondary MDS and provide useful information of therapeutic regimens for subsequent-line treatment of EGFR-mutated lung cancer. To the best of our knowledge, this is the first report of acquired MDS in a lung adenocarcinoma patient.

**Supplementary Information:**

The online version contains supplementary material available at 10.1186/s12920-021-01147-y.

## Background

Myelodysplastic syndromes (MDSs) are clonal stem-cell disorders characterized by ineffective hemopoiesis leading to morphologic dysplasia, peripheral cytopenias, and potentially developing acute myeloid leukemia. The syndromes are more common in older people, with a median age of 65–70 years at diagnosis, and less than 10% of the patients are younger than 50 years of age [1]. MDS can be divided into primary and secondary, and the etiology and pathogenesis of primary MDS are still unclear, while secondary MDS follows treatment with chemotherapy or irradiation. The yearly incidence rate of MDS is approximately 4.5 per 100,000 people in the general population [2], and 10% of the MDS cases are acquired [3]. Secondary MDS has been reported in various solid tumors, such as breast cancer, ovarian cancer, prostate cancer, and lung cancer [4–6]. Compared with primary MDS, secondary MDS presents a higher incidence of chromosomal abnormalities, especially complex (≥ 3) abnormalities, and a worse prognosis [1, 7]. Therefore, secondary MDS has become one of the long-term complications after tumor treatment, which has attracted extensive attention of clinicians.

To date, the diagnosis of MDS is mainly based on blood tests, bone marrow tests, and cytogenetics. The revised WHO classification (WHO 2016) of MDS has included that the presence of an *SF3B1* mutation could be considered as MDS with ringed sideroblasts (MDS-RS) [8]. Sequencing techniques are becoming a complementary diagnostic assay in MDS-RS ever since [9]. With the next-generation sequencing (NGS) analysis, more mutations are found to be involved in either primary or secondary MDS disease, such as epigenetic regulators (*TET2*, *ASXL1*, *DNMT3A*, *IDH1*, *IDH2*, *EZH2*), transcription factors (*ETV6*, *RUNX1*, *TP53*), signal transduction proteins (*CBL*, *JAK2*, *KRAS*, *NRAS*), and genes related to the RNA splicing machinery (*SF3B1*, *SRSF2*, *U2AF1*, *ZRSR2*) [9].

In this report, we described that a non-small cell lung cancer (NSCLC) patient had MDS-associated chromosomal abnormalities via DNA hybrid NGS analysis and was confirmed as MDS by bone marrow tests. Since NGS testing has been widely used for biomarker diagnosis for tumor patients [10], NGS data analysis for chromosomal abnormalities and gene mutations could serve as a supplementary method for the diagnosis of secondary MDS.

## Case presentation

**Fig. 1:**
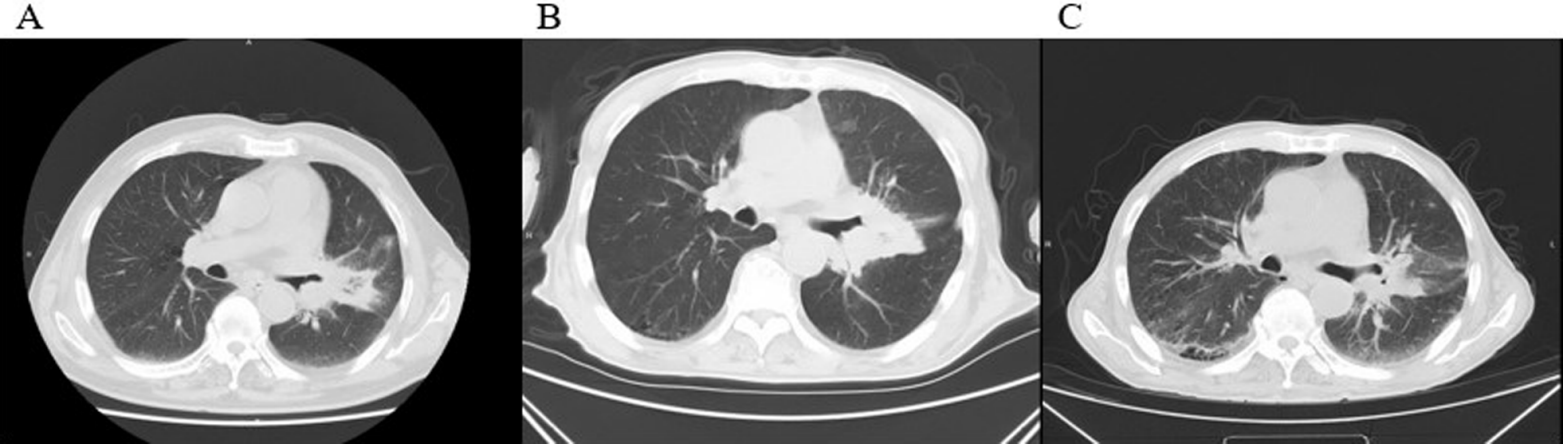
Chest CT scans (mediastinal window). **A** The chest CT scan showed distal obstructive inflammation, the enlargement of the left hilar and mediastinal lymph nodes when the patient was diagnosed with NSCLC. **B** After seven months of gefitinib treatment, the maximal tumor size in the lung lesion did not reduce. **C** After one month of toripalimab and bevacizumab treatment, the tumor was found to be markedly regressed

A 66-year-old male with back pain and cough for two weeks was admitted to the First Hospital of Jiaxing on 8 August 2019. A thoracic computed tomography (CT) scan revealed that the malignant tumor on the left upper lobe was complicated by distal obstructive inflammation, the enlargement of the left hilar and mediastinal lymph nodes, and the multiple bone metastases on 25 August 2019 (Fig. 1A). Immunohistochemical (IHC) results of the posterior iliac bone marrow biopsy specimen showed the positive expression of CD3, CD20, CD34, CD235a, and NPO, and the negative CD61 expression. IHC results of an endoscopic biopsy specimen of the bronchial mucosa on the upper left lobe showed the positive expression of TTF1, CK7, NapsinA, Ki67, CK, and EMA, and the negative expression of CK5/6, P40, CgA, Syn, and CD45 on 6 September 2019. Histopathologic observations showed infiltration of atypia cells in mucosal and fibrous tissues. The detection tools of pathology and cytology included automatic IHC staining (BenchMark XT, Roche, The United States), digital slice scanner, image analysis software (Pannoramic 250, 3DHistech, Hungary), and microscope (Eclipse Ci-S, Nikon, Japan). Finally, the patient was diagnosed with stage IVb lung adenocarcinoma combing with bone metastases.

**Fig. 2:**
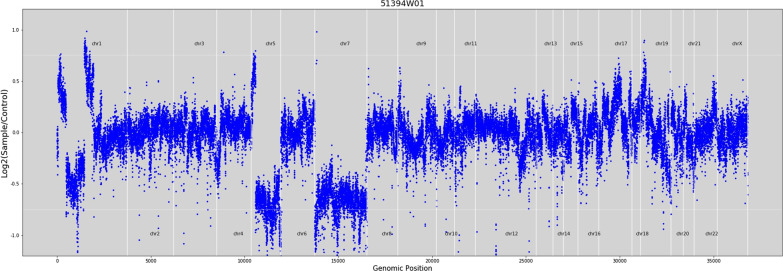
The copy number deletion region on chromosomal 5 and 7 identified by NGS data in this patient

**Fig. 3:**
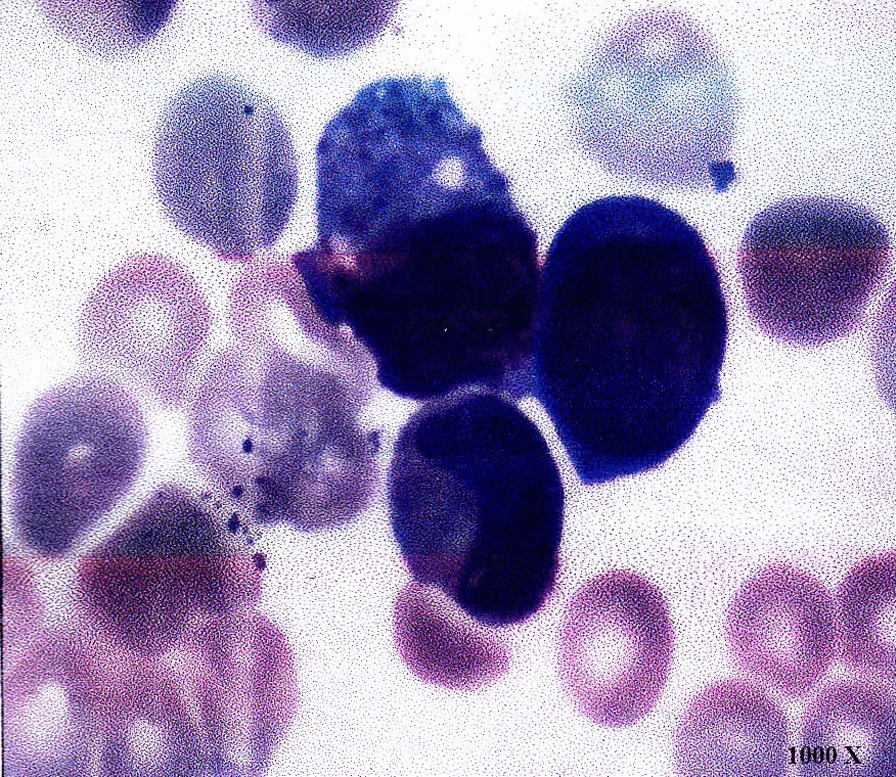
Bone marrow morphology of this case showed the increase of marrow blast. 1000 × represents the multiple of the microscope

To seek potential therapeutic opportunities, the FFPE tissue and control sample (white blood cell) of the patient were detected using a 733-gene NGS panel in a College of American Pathologists (CAP) and Clinical Laboratory Improvement Amendments (CLIA) certificated lab. Sequencing reads were mapped against the hg19/GRCh37 genome, and duplicate reads were removed, followed by variants calling in targeted regions using an in-house developed bioinformatics algorithm. The algorithm utilized a filtering model containing background error correction, strand bias, base quality, mapping quality, short tandem repeat regions, and low-quality mapping ratio 25 [11]. The NGS analysis results indicated that the patient had an EGFR exon 19 p.L747_S752del somatic mutation with an allelic fraction of 73.19% and TP53 p.H179L germline mutation. Besides, the whole chromosome 7 and 5q deletion were detected using NGS-based copy number variation (CNV) analyses (Fig. 2), which were classical abnormalities associated with MDS. According to the clinical inquiry, the patient was a farmer by occupation, which means that he has long been exposed to certain chemicals such as pesticides, fertilizers, and solvents containing benzene. In addition, he smoked for 40 years. Taking all these findings together, we suspected the patient with secondary MDS. The results of the physiological blood indexes and the bone marrow aspiration test showed a decrease of platelet counts and the elevation of myelocyte counts. The proportion of blast was 5%, and the morphology of the cells presented the characteristics of MDS, which further confirmed our speculation (Fig. 3 and Additional file 1: Fig. S1).

**Fig. 4:**
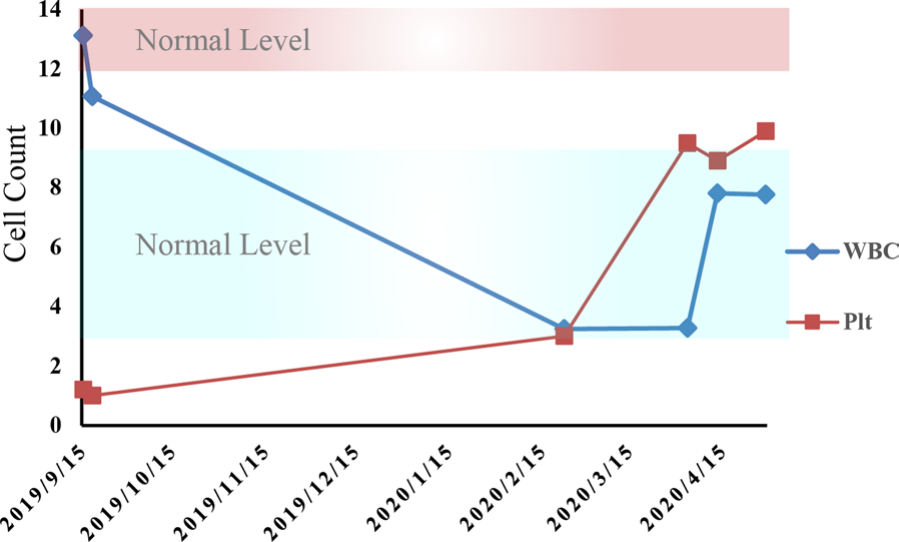
Dynamic changes of white blood cell (WBC) counts and platelet (Plt) counts through the course of therapy. The WBC and platelet count restored near-normal level after treatment. Red background, normal range of platelet count; Blue background, normal range of WBC count

In terms of treatment, the EGFR-positive mutation patient was administered with gefitinib starting from 20 August 2019. Due to decreasing platelet counts, azacitidine was administrated for four courses of treatment. However, the thrombocytopenia had not been significantly improved, and the size of the maximal tumor in the lung lesion did not decrease after seven months’ treatment (Fig. 1B). On 10 April 2020, a pathological report showed the infiltration of poorly differentiated cancer cells into the fibrous tissue. IHC results suggested the neuroendocrine tumor, in which small cell carcinoma and atypical carcinoid tumor accounted for about 40% and 60%, respectively. On 7 April and 28 April 2020, considering positive PD-L1 expression and high tumor mutation burden (TMB) in the tissue sample, the patient was administrated with toripalimab (a PD-1 inhibitor) and bevacizumab. Platelet counts returned to near-normal levels gradually, and the tumor lesion shrank obviously (Fig. 4). It was concluded that the patient reached a partial response (PR) (Fig. 1C). The progression-free survival was four months.

## Discussion and conclusions

The most common techniques for MDS diagnosis are identification of bone marrow morphology and cytogenetic analysis. Since morphology characteristics are subjective and empirical, cytogenetic analysis has been used as a major measurement for MDS diagnosis. Chromosomal anomalies are detected in approximately 50% of patients with primary MDS and 80% of patients with secondary MDS associated with chemotherapy or other toxic agents [12]. With the fast development and wide applications of NGS technologies, many studies have reported that 70–80% of MDS patients carry specific DNA alterations [13].

Here, we reported an NSCLC patient with molecular characteristics of MDS, including TP53 p.H179L mutation, and chromosome 7 and 5q deletion detected using targeted NGS. Deletion of chromosome 5 is one of the most common cytogenetic abnormalities in MDS, accounting for 10–15% of MDS cases [14]. Chromosome 7 deletion with extra cytogenetic aberration is the second most frequent chromosomal abnormality in MDS, associated with poor overall survival and a high transformation rate to leukemia [15]. *TP53* pathogenic mutation mainly develops resistance to apoptosis mechanism. It is reported that *TP53* alterations were in about 10% of MDS patients, predominantly in therapy-related MDS [16]. Based on the common molecular characteristics of MDS by NGS, further cell morphology analysis, such as bone marrow smear, was carried out and confirmed MDS, indicating that NGS detection has additional predictive value in the diagnosis of MDS. Therefore, physicians must pay attention to the NGS results for a better overall treatment plan, regardless of whether patients have already shown specific MDS characteristics or not. Notably, our patient resistant to EGFR-TKI therapy achieved a PR after toripalimab plus bevacizumab, suggesting that immunotherapy combined with anti-angiogenesis therapy may be effective in the subsequent-line treatment of EGFR-mutated lung cancer with positive PD-L1 expression and high TMB [17].

In conclusion, molecular abnormality analysis is valuable in the diagnosis of MDS and pathogenesis research leading to tumorigenesis of MDS patients. Also, our case suggested that NSCLC is one of the cancer types associated with an increased risk of secondary MDS.

## Supplementary Information

**Additional file 1**.** Figure S1**. Other fields of bone marrow morphology.

## Data Availability

All the raw FASTQ data have been deposited in the National Genomics Data Center (NGDC) BioProject database, and the revelant accession number is “PRJCA007440”. The web link to the hg19/GRCh37 genome dataset used in our study is https://hgdownload.cse.ucsc.edu/goldenPath/hg19/bigZips/hg19.fa.gz.

## Abbreviations

MDS: Myelodysplastic syndrome
NSCLC: Non-small cell lung cancer
NGS: Next-generation sequencing
CNV: Copy number variation
PR: Partial response

## References

[1] De Roos AJ, Deeg HJ, Davis S (2007). A population-based study of survival in patients with secondary myelodysplastic syndromes (MDS): impact of type and treatment of primary cancers. Cancer Causes Control.

[2] Jing Y, Shen X, Mei Q, Han W (2015). Spotlight on decitabine for myelodysplastic syndromes in Chinese patients. Onco Targets Ther.

[3] Graubert T (2010). Therapy-related myelodysplastic syndrome: models and genetics. Biol Blood Marrow Transplant.

[4] Kaplan HG, Malmgren JA, Atwood MK (2011). Increased incidence of myelodysplastic syndrome and acute myeloid leukemia following breast cancer treatment with radiation alone or combined with chemotherapy: a registry cohort analysis 1990–2005. BMC Cancer.

[5] Mukherjee S, Reddy CA, Ciezki JP (2014). Risk for developing myelodysplastic syndromes in prostate cancer patients definitively treated with radiation. J Natl Cancer Inst.

[6] Griesinger F, Metz M, Trumper L, Schulz T, Haase D (2004). Secondary leukaemia after cure for locally advanced NSCLC: alkylating type secondary leukaemia after induction therapy with docetaxel and carboplatin for NSCLC IIIB. Lung Cancer.

[7] Bhatia S (2013). Therapy-related myelodysplasia and acute myeloid leukemia. Semin Oncol.

[8] Arber DA, Orazi A, Hasserjian R (2016). The 2016 revision to the World Health Organization classification of myeloid neoplasms and acute leukemia. Blood.

[9] Janusz K, Del Rey M, Abaigar M (2017). A two-step approach for sequencing spliceosome-related genes as a complementary diagnostic assay in MDS patients with ringed sideroblasts. Leuk Res.

[10] Frampton GM, Fichtenholtz A, Otto GA (2013). Development and validation of a clinical cancer genomic profiling test based on massively parallel DNA sequencing. Nat Biotechnol.

[11] Su D, Zhang D, Chen K (2017). High performance of targeted next generation sequencing on variance detection in clinical tumor specimens in comparison with current conventional methods. J Exp Clin Cancer Res.

[12] Haase D, Germing U, Schanz J (2007). New insights into the prognostic impact of the karyotype in MDS and correlation with subtypes: evidence from a core dataset of 2124 patients. Blood.

[13] Haferlach T, Nagata Y, Grossmann V (2014). Landscape of genetic lesions in 944 patients with myelodysplastic syndromes. Leukemia.

[14] Nagata Y, Maciejewski JP (2019). The functional mechanisms of mutations in myelodysplastic syndrome. Leukemia.

[15] Crisà E, Kulasekararaj AG, Adema V (2020). Impact of somatic mutations in myelodysplastic patients with isolated partial or total loss of chromosome 7. Leukemia.

[16] Kita-Sasai Y, Horiike S, Misawa S (2001). International prognostic scoring system and TP53 mutations are independent prognostic indicators for patients with myelodysplastic syndrome. Br J Haematol.

[17] Passaro A, Jänne PA, Mok T (2021). Overcoming therapy resistance in EGFR-mutant lung cancer. Nat Cancer.

