# Supplementary figures and images for: Secondary myelodysplastic syndromes identified via next-generation sequencing in a non-small cell lung cancer patient
Source: BMC Med Genomics. 2021 Dec 20;14:299. doi: 10.1186/s12920-021-01147-y (PMC8691080; doi:10.1186/s12920-021-01147-y)

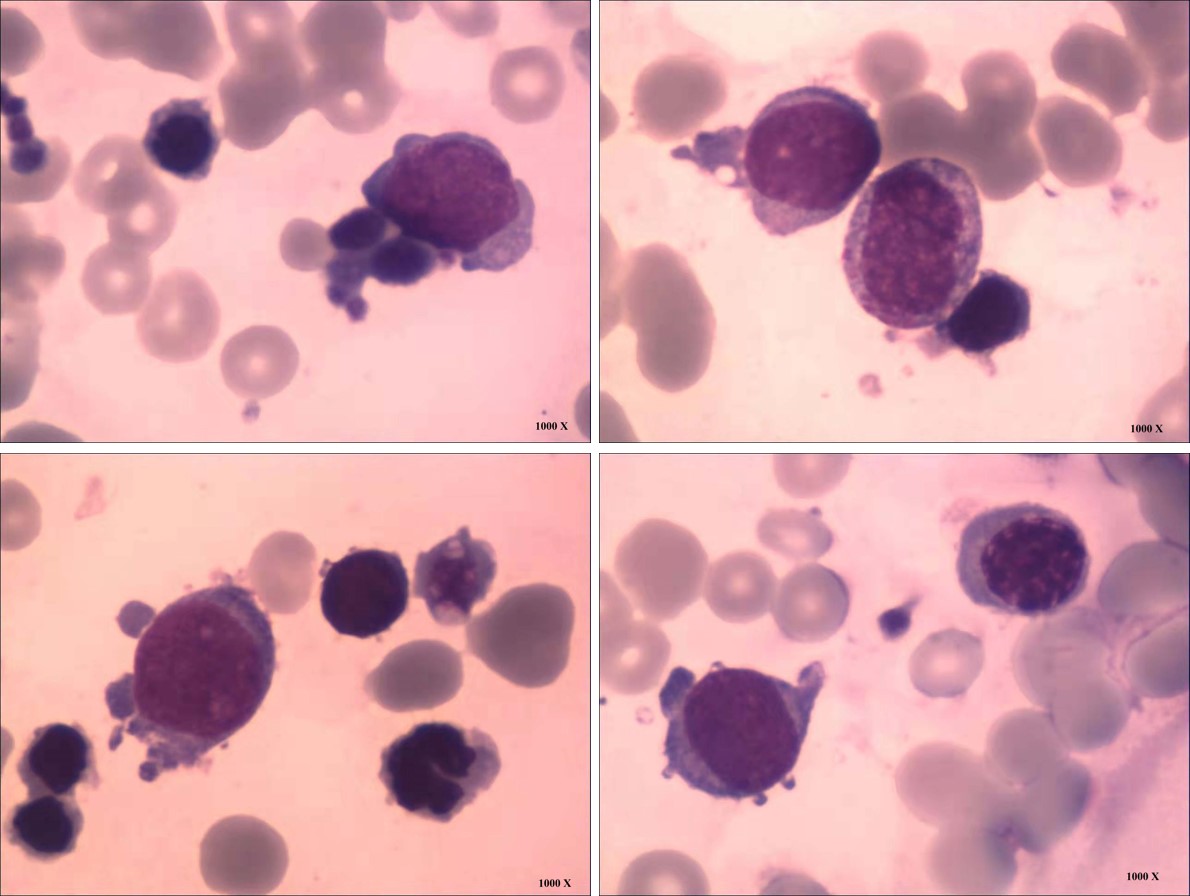

Supplement: Supplementary file 1 — Additional file 1. Figure S1. Other fields of bone marrow morphology. [file 12920_2021_1147_MOESM1_ESM.jpg]
